# The IDF Definition Is Better Suited for Screening Metabolic Syndrome and Estimating Risks of Diabetes in Asian American Adults: Evidence from NHANES 2011–2016

**DOI:** 10.3390/jcm9123871

**Published:** 2020-11-28

**Authors:** Lin Zhu, Cody Spence, Wei Jenny Yang, Grace X. Ma

**Affiliations:** 1 Center for Asian Health, Lewis Katz School of Medicine, Temple University, Philadelphia, PA 19140, USA; tuj24206@temple.edu (W.J.Y.); grace.ma@temple.edu (G.X.M.); 2 Department of Sociology, College of Liberal Art, Temple University, Philadelphia, PA 19122, USA; cspence@temple.edu

**Keywords:** metabolic syndrome, cardiovascular disease, diabetes mellitus, Asian Americans

## Abstract

Objective: extensive effort has been made to better define metabolic syndrome (MetS). Whether current definitions accurately diagnose MetS and predict risk of cardiovascular disease (CVD) or diabetes in diverse ethnic groups remains largely unknown. The objective of this study was to compare the prevalence of MetS and risk of CVD and diabetes among Asian American adults using two MetS definitions, one proposed by the Third Report of the National Cholesterol Education Program Expert Panel on Detection, Evaluation, and Treatment of High Blood Cholesterol in Adults (ATP III) and one by the International Diabetes Federation (IDF). Methods: we obtained a nationally representative sample of 2121 Asian American adults in the noninstitutionalized civilian population of the United States from the National Health and Nutrition Examination Survey (2011–2016). We computed age-adjusted, gender-specific MetS prevalence and each MetS component using ATP III and IDF definitions. Results: based on the IDF definition, MetS prevalence was 39.26% among Asian American men and 39.66% among Asian American women included in the study sample. Based on the ATP III definition, MetS prevalence in our sample was 39.38% among men and 36.11% among women. We found good concordance between the IDF and the ATP III definitions in identifying MetS in Asian American adults. Those with MetS defined only by the IDF definition had significantly higher body mass index (BMI) and waist circumference than those with MetS defined only by the ATP III definition. The IDF definition also better predicted elevated fasting insulin. Conclusions: the IDF definition is more pertinent than the ATP III definition for screening and estimating risk of CVD and diabetes in Asian American adults. Future studies should examine differences in MetS prevalence across Asian ethnic groups to facilitate the development of culturally tailored strategies improve MetS prevention and detection in Asian Americans.

## 1. Introduction

Metabolic syndrome (MetS) is a disease entity characterized by a complex constellation of physiological, biochemical, and metabolic factors [1,2,3,4]—including abdominal obesity, insulin resistance, elevated arterial blood pressure, and dyslipidemia (elevated triglycerides, decreased high-density lipoproteins) [1,2,3,5]. The syndrome has been associated with a five- to seven-fold increase in risk of type 2 diabetes mellitus (T2DM), a three-fold increase in risk of cardiovascular disease (CVD), and a 1.5-fold increase in risk of all-cause mortality [6,7,8,9,10,11]. With approximately one-quarter of the world population suffering from MetS [11], it has become increasingly important to fully examine MetS epidemiology in diverse groups.

Several international organizations and expert groups have attempted to incorporate different parameters to define MetS. The modified National Cholesterol Education Program Adult Treatment Panel III (ATP III) [2] and the International Diabetes Federation (IDF) [12] have become the most widely utilized and compared [13,14,15,16,17,18,19]. While criteria for both ATP III and IDF consider central obesity (defined by waist circumference, with ethnicity- and gender-specific cutoff values), the IDF uses central obesity as a prerequisite for diagnosis, while the ATP III considers central obesity as one component out of several that could be present. As such, it is still controversial which definition is more accurate in detecting CVD risk [20] and for which ethnic groups.

In particular, Asian Americans are often understudied in terms of MetS and CVD prevalence [21,22,23]. Asian Americans represent a growing population in the United States, with distinct body habitus, lipid metabolism, and insulin sensitivity [24,25,26,27,28]. Despite rapidly changing demographics and marked heterogeneity in the community, data on MetS prevalence in Asian Americans are still lacking. Current understanding of cardiometabolic disease burden is distorted by the underrepresentation of Asian Americans in epidemiological surveys [29,30] and the use of limited local and/or regional data [31,32,33].

This study aimed to estimate the sex-specific prevalence of MetS in Asian American adults, according to the ATP III and IDF definitions, and to examine the concordance between the two definitions. The association between MetS and two surrogates for CVD or T2DM—elevated fasting insulin [34,35,36] and elevated uric acid [37,38,39,40,41,42]—was also examined using data from the National Health and Nutrition Examination Survey (NHANES). To the best of our knowledge, this was the first time a nationally representative sample of Asian Americans was used to fully investigate MetS prevalence in this ethnic population.

## 2. Methods

### 2.1. Study Sample

This study is a cross-sectional investigation of non-Hispanic Asian (hereafter referred to as Asian) adults from the National Health and Nutrition Examination Survey (NHANES) 2011–2016. NHANES is one of a series of health-related programs conducted by the Centers for Disease Control and Prevention’s (CDC) National Center for Health Statistics (NCHS). The objectives of NHANES are to monitor trends in the prevalence of selected diseases and to study the relationship between diet, nutrition, and health [43]. NHANES uses a multistage, stratified design to produce a study sample that is representative of the noninstitutionalized civilian resident population in the 50 states and the District of Columbia [43]. The survey consists of two parts. First, survey questionnaires were administered to eligible participants at home, where person-level demographics, health, and nutrition information were collected. Then, participants were invited to visit specially equipped mobile examination centers for a standardized health examination. The survey procedures are detailed elsewhere [43]. The 2011–2016 NHANES oversampled Asian and several other subpopulations to increase the precision of estimates for these groups. To facilitate the oversampling of the Asian population, NHANES provided survey materials and a promotional video in traditional and simplified Mandarin, Korean, and Vietnamese [44]. This study specifically used the subsample of the participants who were 18 years old or older and who self-identified as non-Hispanic Asian. This selection generated a sample of 2121 individuals. 

### 2.2. Measures

#### 2.2.1. Metabolic Syndrome

The two definitions of MetS are presented in Table 1. Under the modified ATP III definition [2], an individual is considered to have MetS if he or she has at least three of the following five factors: (1) central obesity (waist circumference > 102 cm in men or > 88 cm in women); (2) raised triglycerides (≥150 mg/dL) or specific treatment for this lipid abnormality; (3) reduced high-density lipoproteins (HDL) cholesterol (<40 mg/dL in males, <50 mg/dL in females) or specific treatment for this lipid abnormality; (4) raised blood pressure (blood pressure ≥ 130/85 mm Hg) or treatment of previously identified hypertension; and (5) raised fasting plasma glucose (≥100 mg/dL) or previously diagnosed T2DM. Under the IDF definition [12], an individual is deemed to have MetS if he or she has central obesity (waist circumference ≥ 90 cm for South and East Asian men and ≥80 cm for South and East Asian women, with ethnicity specific values, assumed if BMI is >30 kg/m^2^), plus any two of the following four factors: (1) raised triglycerides (≥150 mg/dL) or specific treatment for this lipid abnormality; (2) reduced HDL cholesterol (<40 mg/dL in males, <50 mg/dL in females) or specific treatment for this lipid abnormality; (3) raised blood pressure (blood pressure ≥ 130/85 mm Hg) or treatment of previously identified hypertension; and (4) raised fasting plasma glucose (≥100 mg/dL) or previously diagnosed T2DM. The differences between the two definitions are that the IDF considers central obesity a prerequisite for MetS diagnosis and uses lower thresholds of waist circumference for South and East Asian men and women, whereas the ATP III definition does not use this prerequisite or threshold.

#### 2.2.2. CVD/T2DM Surrogates

Fasting insulin and uric acid were used as surrogates for CVD or T2DM. Elevated fasting insulin was defined as fasting insulin higher than 48 pmol/L (8 µIU/L) [45]. Elevated uric acid level was defined as uric acid higher than 6 mg/dL for women and 7 mg/dL for men [46]. 

#### 2.2.3. Demographic Characteristics

We adjust for gender (man or woman), age (in years), marital status (currently married or not), education (high school or below; college or some college; or graduate degree), poverty level (ratio of annual family income to federal poverty line), physical activity level (in four categories based on metabolic equivalent score), and alcohol consumption (lifetime abstainer; former drinker; non-excessive current drinker; or excessive current drinker), and smoking status (current smoker or not).

### 2.3. Statistical Analysis

We applied the appropriate sample weights according to the National Center for Health Statistics guidelines to account for the complex survey design, the oversampling of Asian Americans, and the fact that the data on fasting glucose were collected on a subsample of the population [47]. We used the *svy* command in Stata to apply the weights. To handle missing values on laboratory and examination variables, we employed multiple imputation (MI), a commonly used, model-based approach for dealing with missing data. Using this technique, each missing value was replaced with multiple imputed values to create multiple complete datasets. Using specially designed commands in Stata 16, each dataset is analyzed separately, and the results are combined to obtain valid statistical inferences [48].

We computed age-adjusted, gender-specific MetS prevalence and each MetS component using ATP III and IDF definitions. We also calculated the age-adjusted, average values of several clinical characteristics among two groups: those who were identified as having MetS by the ATP III definition but not the IDF definition, and those identified as having MetS by the IDF definition but not the ATP III definition. We used the age distribution in the US 2010 Census to standardize the prevalence and means of MetS and various clinical characteristics. We used *t*-tests to examine differences in the clinical characteristics between the two groups. All prevalence and means were presented as a percentage or mean with 95% confidence intervals (CIs).

In addition, we evaluated the agreement between the two MetS definitions with the percentage of concordant cases, sensitivity, and specificity with the kappa index. The sensitivity of the IDF definition was calculated as the percentage of the ATP III-defined MetS cases that were also identified as MetS by the IDF definition. The specificity of the IDF definition was calculated as the percentage of the non-MetS cases under the ATP III definition that were also identified as non-MetS by the IDF definition.

The concordance between the two definitions is considered excellent for a Kappa index value greater than 0.80, good for a value between 0.61 and 0.80, moderate for a value between 0.41 and 0.60, and weak for a value of 0.40 or below. Because the *mi estimate* command does not support the command to generate the kappa index, we calculated the statistic by subtracting the hypothetical probability of chance agreement from the relative observed agreement between the two definitions, then dividing it by the hypothetical probability of chance agreement.

We used logistic regression to calculate the odds ratios (ORs) and their 95% CIs. In the logistic regression models, we controlled for gender, age, marital status, education, poverty level, physical activity level, alcohol consumption, and smoking status. A *p*-value of 0.05 or below was considered statistically significant. All data analyses were conducted in Stata 16 [49].

## 3. Results

A total of 2121 Asian American subjects (1040 men and 1081 women) were included in the study sample. The average age was 44.18 (95% CI: 42.80–45.56). Table 2 shows the age-adjusted prevalence of MetS and five components under two definitions. By either definition, more than one-third of the Asian American adults, regardless of gender, had MetS. By the ATP III definition, the MetS prevalence was 39.38% (35.97–42.79%) in men and 36.11% (33.32–38.90%) in women. The prevalence of central obesity (defined as waist circumference > 102 cm in men or > 88 cm in women) was 14.31% (11.98–16.64%) in men and 36.71% (34.34–39.09%) in women.

By the IDF definition, the MetS prevalence was 39.26% (35.91%–42.60%) in men and 39.66% (36.93–42.39%) in women. Compared to the rates by the ATP III definition, the MetS prevalence by the IDF definition was similar in men but slightly higher in women. The prevalence of central obesity (defined as waist circumference > 90 cm in men or > 80 cm in women) was 52.36% (49.12–55.59%) in men and 66.77% (63.99–69.54%) in women. Compared to the rates by the ATP III definition, the central obesity rates by the IDF definition were drastically higher in both men and women. 

For the other four components of MetS, the ATP III and IDF definitions have the same threshold, hence the same rates by the two definitions. The prevalence of raised serum triglycerides was 54.00% (51.07–56.94%) in men and 41.01% (37.96–44.05%) in women. The prevalence of reduced serum HDL was 37.90% (34.75–41.38%) in men and 39.26% (35.74–42.77%) in women. The prevalence of raised blood pressure was 39.63% (36.57–42.68%) in men and 33.62% (31.31–35.93%) in women. The prevalence of raised fasting plasma glucose was 60.29% (56.22–64.36%) in men and 42.37% (38.01–46.73%) in women. 

Table 3 shows MetS status by the two definitions. The sensitivity of the IDF definition was 86, which means that the IDF definition identified MetS among 86% of those with ATP III-defined MetS. The specificity was 88, which means that the IDF definition identified non-MetS (i.e., normal) among 88% of those that were identified as non-MetS by the ATP III definition. The kappa index was 0.73, which indicates good concordance between the two definitions.

Table 4 shows the age-adjusted means of seven clinical characteristics among two groups. We used t-test to compare the mean values of each characteristic by two groups: subjects who were identified as having MetS by the ATP III definition but not the IDF definition (i.e., Group 1) and those who were identified as having MetS by the IDF definition but not the ATP III definition (i.e., Group 2). Among men, Group 2 had a significantly higher mean body mass index (BMI; *p* < 0.001) and a higher mean waist circumference (*p* < 0.001), than did Group 1. The results were similar among women. For both men and women, we did not find significant differences between Group 1 and Group 2 in mean triglycerides, HDL, systolic or diastolic blood pressure, or fasting plasma glucose.

Table 5 shows the results of logistic regression on two CVD/T2DM surrogates: elevated fasting insulin and elevated uric acid. We found that MetS by the ATP III definition (OR = 4.27, 95% CI: 3.14–5.80) and IDF definition (OR = 4.59, 95% CI: 3.35–6.28) were both independently and significantly associated with elevated fasting insulin, but not with elevated uric acid. We then divided the study sample into four subgroups of MetS status: no MetS, MetS defined by both definitions, MetS defined only by ATP III, and MetS defined only by IDF. Logistic regression results showed that two groups, those with MetS defined by both definitions (OR = 6.07, 95% CI: 4.20–8.78) and those with MetS defined only by IDF (OR = 2.54, 95% CI: 1.52–4.23) were significantly more likely than the reference group (those without MetS) to have elevated fasting insulin. We found no significant differences in the likelihood of having elevated uric acid across the four subgroups. For all logistic regression models, we adjusted for potential confounders, including gender, age, marital status, education, poverty level, physical activity level, alcohol consumption, and smoking status.

## 4. Discussion

To the best of our knowledge, this is the first study to estimate MetS prevalence using both the ATP III and the IDF definitions in a nationally representative sample of Asian American adults. This study has four main findings. First, MetS is common in Asian Americans, regardless of the definition used. Under either ATP III or IDF definitions, more than one-third of Asian American adults had MetS. This finding adds to the growing literature on the increasing burden of heart disease and diabetes in this population and debunks the stereotypes, or rather, myths, of Asian Americans being “lean and healthy” and “less prone to heart diseases” [30,50]. IDF-defined MetS prevalence in this study population was similar in men (39.26% vs. 39.38%) and slightly higher in women (39.66% vs. 36.11%), when compared to prevalence by the ATP III definition. Previous studies conducted in Asian countries have reported a wide range of MetS prevalence, with the rate ranging from 29–45% in India [51,52,53,54], 14–26% in the Phillipines [55,56,57], 9–29% in China [58,59,60,61,62,63,64,65], 7–31% in South Korea [66,67,68,69], and 4–16% in Vietnam [70,71,72,73]. The prevalence rates in this study were similar to those reported in India, but higher than those reported in the Philippines, China, South Korea, and Vietnam. This points to the need to examine MetS prevalence within each detailed Asian ethnic group to identify high-risk groups. Furthermore, potential differences in MetS risks between Asian Americans in the United States and populations of Asians in their native countries are also worth examining, as such analyses could shed light on epidemiological mechanisms associated with MetS.

Second, we found that there was good concordance between the IDF and ATP III definitions in identifying MetS in Asian American adults. There was a high level of agreement in diagnosis between the two definitions, as indicated by the sensitivity, specificity, and the kappa index. Previous studies have generated conflicting results. While some studies found good concordance between the two definitions in South Korea [74], China [64,65], and Taiwan [75], other studies found low concordance in diverse populations [63,76,77]. These inconsistencies in the literature suggest significant heterogeneity in overall obesity and central obesity by ethnicity. The epidemiology of overall and central obesity and the association of these obesity factors with cardiometabolic risk profiles have yet to be thoroughly examined in detail in Asian ethnic groups, especially in Asian American populations.

Third, regardless of sex, those with MetS defined only by the IDF definition had significantly higher BMI and waist circumference than those with MetS defined only by the ATP III definition, despite the lower cutoff points of waist circumference used by the IDF definition, which was consistent with findings from a previous study on the Hong Kong Chinese working population [78]. The differences between the two groups in this study are likely due to the fact that central obesity is a prerequisite for diagnosis in the IDF definition [63]. In other words, the IDF definition included more people with central obesity when diagnosing MetS. A higher emphasis on central obesity with lower cutoff points in the context of the evaluation of cardiometabolic risk profiles for South and East Asian individuals [31,32,79], especially women, has been embraced by researchers and health professionals in recent years. Research has shown that individuals of South and East Asian heritage, especially women, have greater abdominal and visceral adiposity than Caucasians with similar BMI [80,81]. Greater abdominal and visceral adiposity is consistently found to be associated with elevated cardiometabolic risks, which may explain the “lean yet unhealthy” myth [82,83], particularly in Asian populations, a topic that has received increasing attention from researchers in recent decades [84,85].

The fourth main finding of this study is that the IDF definition better predicted elevated fasting insulin, a risk factor for diabetes, than did the ATP III definition. Previous studies on Asian populations have generated conflicting findings. While one study conducted in China found that the IDF definition was better at detecting cardiovascular risk [86], two other studies conducted in China [64] and South Korea [73] reported that the ATP III definition was more closely associated with CVD. These inconsistencies once again point to the need to examine different definitions of MetS and their association with cardiometabolic risks within each specific ethnic group. We did not find MetS defined by either definition to be associated with elevated uric acid.

This study is not without limitations. A primary limitation is that we estimated the MetS prevalence in aggregated Asian American adults. Given the potentially significant heterogeneity in MetS prevalence and cardiovascular risk profile by ethnicity and age group, more analyses are needed that include detailed ethnic groups, the elderly, and children. Another limitation is that we did not compare the agreement between the ATP III and IDF definitions with the World Health Organization (WHO) MetS definition [87]. The modified WHO definition identifies an individual as having MetS if he or she has diabetes or impaired glucose tolerance (2 h post-oral glucose load plasma glucose ≥ 140 mg/dL), and two of the four following conditions: (1) BMI > 30 kg/m^2^ or waist-to-hip ratio > 0.9 in men and waist-to-hip ratio > 0.85 in women; (2) dyslipidemia, defined as triglycerides > 150 mg/dL or HDL < 35 mg/dL in men and < 39 mg/dL in women; (3) raised blood pressure (blood pressure ≥ 140/90 mm Hg) or treatment of previously identified hypertension; (4) microalbuminuria (i.e., urinary albumin excretion rate ≥ 200 µgm/minute or albumin/creatine ratio ≥ 30 μgm/mg). While the WHO definition has been used in epidemiological studies in different countries, it is more complex than the IDF definition because it includes measurement of microalbuminuria and plasma insulin levels. This makes it more difficult to apply the WHO definition in the primary care or community setting [63]. Furthermore, this study is cross-sectional in nature, which limited our ability to make causal inference. Longitudinal analysis is necessary to examine the temporal, causal relationship between MetS and elevated fasting insulin and other cardiometabolic risks. 

In conclusion, we found that more than one-third of Asian American adults have MetS. The IDF definition is more pertinent than the ATP III definition for screening and estimating cardiovascular risk in Asian American adults. Future studies should further examine the differences of MetS prevalence across detailed Asian ethnic groups. Public health efforts are also needed to design culturally relevant strategies for better prevention, detection, and linkage to care for MetS in Asian American communities.

## Figures and Tables

**Table 1:** MetS as defined by ATP III and IDF criteria.

ATP III Definition (2005)		IDF Definition (2005)	
Criteria: any three or more of the following five factors:		Criteria: central obesity (defined as waist circumference ≥ 90 cm for South and East Asian men and ≥ 80 cm for South and East Asian women, with ethnicity specific values, assumed if BMI is >30 kg/m^2^)	
Central obesity, defined as waist circumference > 102 cm in men or > 88 cm in women		plus, any two of the following four factors:	
Raised triglycerides	≥150 mg/dL	Raised triglycerides	≥150 mg/dL
	or specific treatment for this lipid abnormality		or specific treatment for this lipid abnormality
Reduced HDL cholesterol	<40 mg/dL in males <50 mg/dL in females	Reduced HDL cholesterol	<40 mg/dL in males <50 mg/dL in females
	or specific treatment for this lipid abnormality		or specific treatment for this lipid abnormality
Raised blood pressure	≥130/85 mm Hg	Raised blood pressure	≥130/85 mm Hg
	or treatment of previously identified hypertension		or treatment of previously identified hypertension
Raised fasting plasma glucose	≥100 mg/dL	Raised fasting plasma glucose	≥100 mg/dL
	or previously diagnosed T2DM		or previously diagnosed T2DM

ATP III = Third Report of the National Cholesterol Education Program Expert Panel on Detection, Evaluation, and Treatment of High Blood Cholesterol in Adults; IDF = International Diabetes Federation; MetS = metabolic syndrome; HDL = high-density lipoproteins; T2DM = type 2 diabetes mellitus.

**Table 2:** Age-adjusted prevalence of MetS and five components in study subjects, by gender (N = 2121).

Gender		ATP III	IDF
Men	MetS	39.38 (35.97–42.79)	39.26 (35.91–42.60)
	Central obesity	14.31 (11.98–16.64)	52.36 (49.12–55.59)
	Raised serum triglycerides	54.00 (51.07–56.94)	54.00 (51.07–56.94)
	Reduced serum HDL	37.90 (34.75–41.38)	37.90 (34.75–41.38)
	Raised blood pressure	39.63 (36.57–42.68)	39.63 (36.57–42.68)
	Raised fasting plasma glucose	60.29 (56.22–64.36)	60.29 (56.22–64.36)
Women	MetS	36.11 (33.32–38.90)	39.66 (36.93–42.39)
	Central obesity	36.71 (34.34–39.09)	66.77 (63.99–69.54)
	Raised serum triglycerides	41.01 (37.96–44.05)	41.01 (37.96–44.05)
	Reduced serum HDL	39.26 (35.74–42.77)	39.26 (35.74–42.77)
	Raised blood pressure	33.62 (31.31–35.93)	33.62 (31.31–35.93)
	Raised fasting plasma glucose	42.37 (38.01–46.73)	42.37 (38.01–46.73)

The prevalence rates were standardized using the age distribution in the US 2010 Census. Abbreviation: ATP III = Third Report of the National Cholesterol Education Program Expert Panel on Detection, Evaluation, and Treatment of High Blood Cholesterol in Adults; IDF = International Diabetes Federation; MetS = metabolic syndrome; CI = confidence interval; HDL = high-density lipoproteins.

**Table 3:** Sensitivity, specificity, and kappa index of the IDF definition for detecting MetS under the ATP III definition.

MetS Definition	IDF Definition		
	Sensitivity	Specificity	Kappa index
ATP III definition	86	88	0.73

Abbreviation: ATP III = Third Report of the National Cholesterol Education Program Expert Panel on Detection, Evaluation, and Treatment of High Blood Cholesterol in Adults; IDF = International Diabetes Federation; MetS = metabolic syndrome.

**Table 4:** Comparison of age-adjusted clinical characteristics of subjects with IDF-defined MetS vs. subjects with ATP III-defined MetS, by gender.

Gender	Clinical Characteristics	Group 1 ATP III-Defined	Group 2 IDF-Defined	*p*
		Mean (95% CI)	Mean (95% CI)	
Men	Body mass index	23.26 (22.52–24.00)	26.77 (26.18–27.36)	<0.001
	Waist circumference	85.12 (83.74–86.51)	95.78 (94.90–96.66)	<0.001
	Serum triglycerides	245.73 (212.68–278.77)	206.30 (175.65–236.95)	ns
	Serum HDL	42.58 (39.99–45.17)	45.46 (42.85–48.08)	ns
	Systolic blood pressure	124.42 (120.62–128.21)	126.73 (123.34–130.11)	ns
	Diastolic blood pressure	74.00 (71.01–77.00)	75.65 (72.91–78.38)	ns
	Fasting plasma glucose	115.43 (107.43–123.44)	108.81 (103.08–114.54)	ns
Women	Body mass index	21.16 (20.23–22.08)	23.74 (23.12–24.36)	<0.001
	Waist circumference	75.83 (73.64–78.03)	83.44 (82.65–84.22)	<0.001
	Serum triglycerides	241.50 (180.47–302.52)	165.25 (134.44–196.07)	ns
	Serum HDL	49.42 (42.64–56.21)	55.38 (51.23–59.63)	ns
	Systolic blood pressure	121.78 (116.76–126.80)	127.34 (116.75–137.94)	ns
	Diastolic blood pressure	71.59 (67.85–75.33)	72.98 (63.17–82.78)	ns
	Fasting plasma glucose	107.52 (94.92–120.12)	104.21 (97.51–110.90)	ns

Group 1: subjects identified having MetS by the ATP III definition but not the IDF definition; Group 2: subjects identified having MetS by the IDF definition but not the ATP III definition. All mean values were standardized using the age distribution in the US 2010 Census. Abbreviations: ATP III = Third Report of the National Cholesterol Education Program Expert Panel on Detection, Evaluation, and Treatment of High Blood Cholesterol in Adults; IDF = International Diabetes Federation; MetS = metabolic syndrome; CI = confidence interval; TG: triglyceride; HDL: high-density; ns = not significant.

**Table 5:** Odds ratios and 95% confidence intervals (CI) of CVD/T2DM surrogates for MetS as defined by the ATP III and IDF criteria.

	Elevated Fasting Insulin OR (95% CI)	Elevated Uric Acid OR (95% CI)
**MetS by definition**		
ATP III	4.27 (3.14–5.80) ***	1.19 (0.77–1.84)
IDF	4.59 (3.35–6.28) ***	1.30 (0.89–1.88)
**Subgroups of MetS (ref: no MetS)**		
MetS defined by both	6.07 (4.20–8.78) ***	1.29 (0.82–2.04)
MetS only by ATP III	1.75 (0.98–3.13)	1.17 (0.60–2.28)
MetS only by IDF	2.54 (1.52–4.23) **	1.44 (0.79–2.61)

Adjusted for gender, age, marital status, education, poverty level, physical activity level, alcohol consumption, and smoking status. Abbreviations: ATP III = Third Report of the National Cholesterol Education Program Expert Panel on Detection, Evaluation, and Treatment of High Blood Cholesterol in Adults; IDF = International Diabetes Federation; MetS = metabolic syndrome; CVD = cardiovascular disease; T2DM = type 2 diabetes mellitus; OR = odds ratio; CI = confidence interval; ** *p* < 0.1; *** *p* < 0.001.
